# Decipher RNA isoform combinations from minigene splicing assays and massive parallel sequencing with MAGIC

**DOI:** 10.1093/bioinformatics/btaf525

**Published:** 2025-09-18

**Authors:** Camille Aucouturier, Nicolas Goardon, Laurent Castéra, Alexandre Atkinson, Thibaut Lavolé, Angélina Legros, Agathe Ricou, Flavie Boulouard, Sophie Krieger, Raphaël Leman

**Affiliations:** Laboratoire de biologie et de génétique du cancer, Département de Biopathologie, Centre François Baclesse, Caen 14000, France; Inserm U1245, Cancer and Brain Genomics, FHU G4 Genomics, Normandie University, Rouen 76183, France; Normandie Univ, UNICAEN, Caen 14000, France; Laboratoire de biologie et de génétique du cancer, Département de Biopathologie, Centre François Baclesse, Caen 14000, France; Inserm U1245, Cancer and Brain Genomics, FHU G4 Genomics, Normandie University, Rouen 76183, France; Laboratoire de biologie et de génétique du cancer, Département de Biopathologie, Centre François Baclesse, Caen 14000, France; Inserm U1245, Cancer and Brain Genomics, FHU G4 Genomics, Normandie University, Rouen 76183, France; Laboratoire de biologie et de génétique du cancer, Département de Biopathologie, Centre François Baclesse, Caen 14000, France; Laboratoire de biologie et de génétique du cancer, Département de Biopathologie, Centre François Baclesse, Caen 14000, France; Laboratoire de biologie et de génétique du cancer, Département de Biopathologie, Centre François Baclesse, Caen 14000, France; Laboratoire de biologie et de génétique du cancer, Département de Biopathologie, Centre François Baclesse, Caen 14000, France; Inserm U1245, Cancer and Brain Genomics, FHU G4 Genomics, Normandie University, Rouen 76183, France; Laboratoire de biologie et de génétique du cancer, Département de Biopathologie, Centre François Baclesse, Caen 14000, France; Inserm U1245, Cancer and Brain Genomics, FHU G4 Genomics, Normandie University, Rouen 76183, France; Laboratoire de biologie et de génétique du cancer, Département de Biopathologie, Centre François Baclesse, Caen 14000, France; Inserm U1245, Cancer and Brain Genomics, FHU G4 Genomics, Normandie University, Rouen 76183, France; Normandie Univ, UNICAEN, Caen 14000, France; Laboratoire de biologie et de génétique du cancer, Département de Biopathologie, Centre François Baclesse, Caen 14000, France; Inserm U1245, Cancer and Brain Genomics, FHU G4 Genomics, Normandie University, Rouen 76183, France

## Abstract

**Summary:**

Functional testing of RNA using minigene splicing assays is increasingly being realized to demonstrate the effects of variants on splicing. In complex cases, variant pathogenicity is assessed by Sanger sequencing, which can be time consuming and may be replaced by short read sequencing. Moreover, strategies based on long read sequencing of the amplified minigene construct are promising and allow the isoforms to be fully characterized. We introduce MAGIC, a user-friendly tool that first generates the artificial construction genome files required to then perform alignment, assembly and annotation of the isoforms obtained by either short or long read minigene splicing assay sequencing.

**Availability and implementation:**

MAGIC is available at https://github.com/LBGC-CFB/MAGIC. Zenodo DOI: 10.5281/zenodo.17052752.

## 1 Background

**Figure 1.**
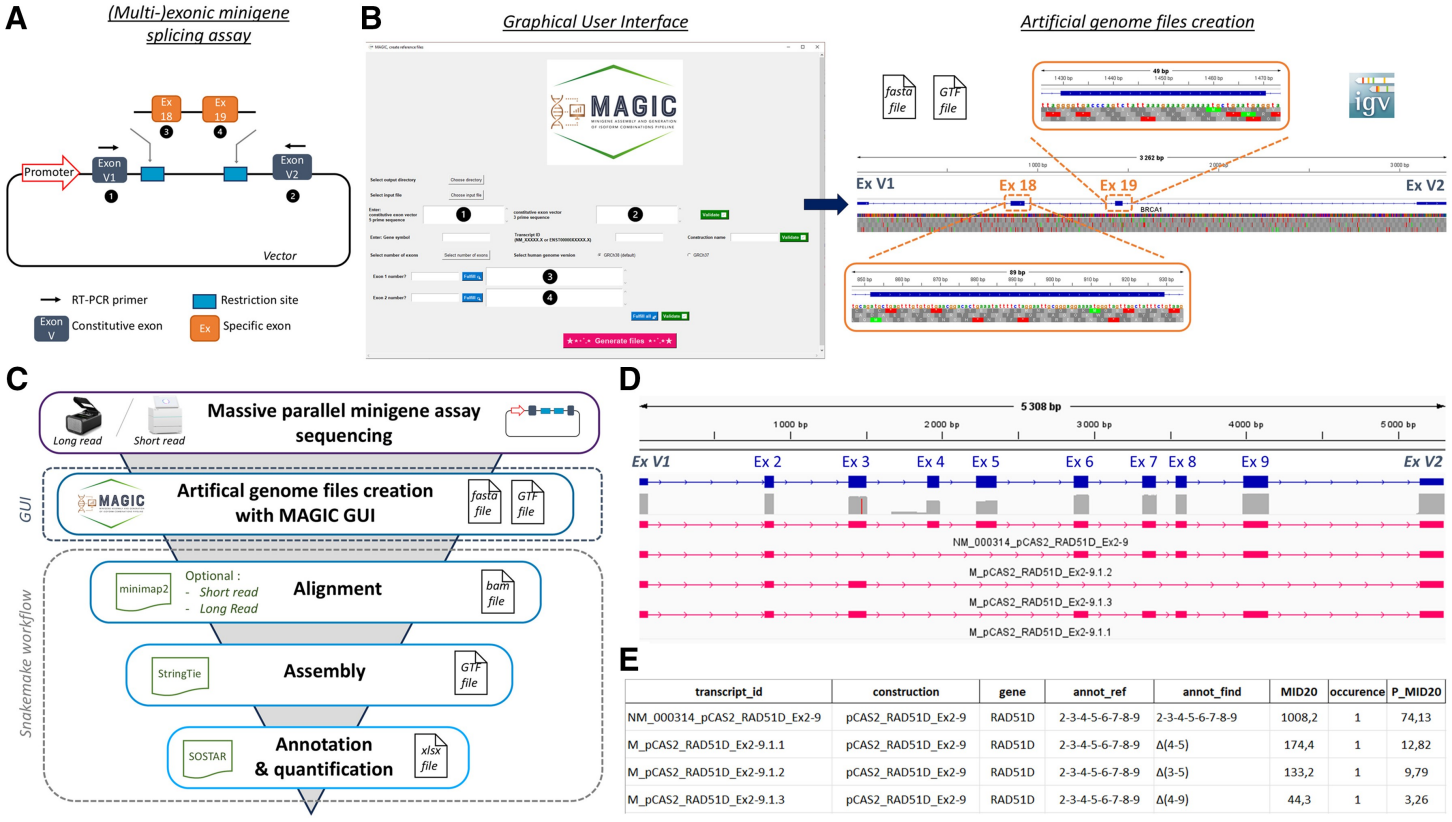
MAGIC workflow. (A) Example of a multi-exonic minigene splicing construction with two specific exons. (B) Example of the MAGIC GUI and its files loaded onto the IGV software (Robinson *et al.* 2011). (C) Overview of the MAGIC pipeline included the GUI. (D) Sequencing results obtained for the multi-exonic minigene construction spanning exons 2–9 of the RAD51D gene in the splicing vector pCAS2. Bam and GTF files are loaded onto IGV software. (E) Final annotated table generated by MAGIC.

In light of the proportion of variants that affect RNA splicing (Dufner-Almeida *et al.* 2019, Truty *et al.* 2021), a significant number of bioinformatics tools have been developed with the objective of predicting the impact of variants on RNA (Leman *et al.* 2022, Canson *et al.* 2023). While these tools facilitate the prioritization of RNA analyses to demonstrate such an effect, RNA analyses are inherently time-consuming and may present a challenge to reach definitive conclusions (Buisine *et al.* 2025). One possible approach for conducting such analyses is through functional testing, which includes various RNA-based techniques. Among these, well-validated *in vitro* minigene assays (Gaildrat *et al.* 2010) are particularly useful for assessing the impact of variants on splicing. High throughput splicing reporter assays with short-read sequencing has enabled greater efficiency (Adamson *et al.* 2018). Moreover, long read RNA sequencing allows the comprehensive characterization of transcripts, thereby facilitating the identification of aberrant ones (Aucouturier *et al.* 2024). The combination of long read sequencing and multi-exonic minigene assays (Fig. 1A) demonstrated the efficacy of this approach in detecting the high complexity of aberrant splicing (Tamayo *et al.* 2023). This strategy requires the definition of an artificial genome comprising the cloned sequence of interest and the minigene expression vector, which is used for bioinformatic analyses. Therefore, we present a novel tool that allows the generation of reference files and its associated pipeline for aligning, assembling and annotating the isoforms obtained from short or long read minigene assay sequencing.

## 2 Description of MAGIC

MAGIC for “Minigene Assembly and Generation of Isoform Combinations tool” is a tool comprised of two distinct components. The first component is a graphical user interface (GUI) that has been written in Python using the Tkinter module. The software has been developed for the purpose of enabling users to create the necessary files of an artificial genome representing the minigene construction used, regardless of their level of bioinformatics expertise (Fig. 1B). The GUI first prompts the user to upload the sequence file containing the complete minigene construction. This sequence file should be submitted in either the Fasta or GenBank format. The user is requested to provide also the sequences of the constitutive exon of the vector located on either side of the cloned sequence of interest. The artificial genome corresponding to the amplified splicing vector is delineated by the given sequences and is created in Fasta format. The user is then required to specify a gene symbol, a transcript name and a construction name. These elements are mandatory for the generation of the GTF file, which delineates the features of the construction. Finally, the user is requested to provide the number of cloned exons, with their corresponding number and their sequence. To facilitate this step, an automated retrieval option is available: when a gene symbol and transcript ID are provided (NM or ENST), the corresponding exon sequences can be automatically populated via a query to the Ensembl database. This feature is currently available for the human genome only (GRCh37 and GRCh38), the fields remain editable to allow manual correction if needed. The constitutive exons are considered and defined as Untranslated Region (UTRs) in the GTF file created, while the specific exons are defined as exons. Their coordinates are calculated relative to the amplified splicing vector.

The second component corresponds to a pipeline based on the previously published SOSTAR pipeline (Aucouturier *et al.* 2024). The Snakemake workflow has been modified to allow users to align data using minimap2 (v2.29-r1283) with specific options for short read or long read sequencing data (Fig. 1C). The alignment was performed on the artificial genome representing the amplified splicing minigene construct previously generated by the GUI. The isoforms obtained from the minigene were then assembled using the StringTie tool (v3.0.0), guided by the GTF file of the artificial genome. Finally, the assembled isoforms were annotated using the SOSTAR nomenclature (Fig. 1, available as supplementary data at *Bioinformatics* online) based on the exons of the cloned interest sequence from the minigene construct. A final table corresponding to all assembled isoforms obtained from the minigene was produced in the xlsx format. Each row corresponds to an isoform, information about these isoforms is contained in the different columns such as the following. The identifier of transcript (transcript_id), the name of the construction (construction), the gene symbol (gene), the specific exons cloned in the minigene (annot_ref), the SOSTAR annotation (annot_find), the expression value computed by StringTie (patient custom number), the number of occurrences in the patient cohort (occurrence), and the relative proportion of the isoform compared to all isoforms obtained from the same construct (patient custom number with “P_” prefix).

## 3 Usage

To demonstrate the utility of MAGIC, we applied it to a multi-exonic minigene construction spanning exons 2–9 of the *RAD51D* gene in the splicing vector pCAS2 (Gaildrat *et al.* 2010) adapted from (Bueno-Martínez *et al.* 2021). Full-length isoforms derived from this minigene were amplified and sequenced on the PrometION P2 Solo nanopore sequencing device using the Ligation Sequencing Kit V14 (SQK-LSK114). Using MAGIC GUI, the artificial genome files were generated by filling in the constitutive exon sequences and the eight specific exon sequences. The transcript name was defined as NM_002878, construction name as pCAS2_RAD51D_Ex2-9, and gene symbol as *RAD51D* (Figs 2 and 3, available as supplementary data at *Bioinformatics* online). The files were then processed in the MAGIC pipeline (Fig. 1D) with the minimap2 aligner tool, and the final table was generated (Fig. 1E). A total of four isoforms were annotated by the pipeline. They corresponded to the full-length isoform comprising the 8 specific exons and the constitutive exons, and three other isoforms with the alternative splicing events Δ(4–5), Δ(3–5) or Δ(4–9). These results were consistent with those previously obtained by (Bueno-Martínez *et al.* 2021). We compare the results obtained using the MAGIC technique with those obtained using the current technique of capillary electrophoresis of RT-PCR amplicons (Fig. 4, available as supplementary data at *Bioinformatics* online). The three main isoforms were retrieved. The Δ(4–9) isoform was detected but with an expression level similar to the background noise. The relative expression of the isoforms was correlated (correlation coefficient 0.9993) with these two techniques.

## 4 Conclusion

To ensure user accessibility, MAGIC has a user-friendly graphical user interface (GUI) that allows users to create and validate the artificial genome files representative of the minigene construction they have designed. The pipeline then helps to describe the full structure of the isoforms derived from the minigenes sequenced on either short or long reads. It opens up new possibilities for the analysis of multi-functional splicing assay.

## Supplementary Material

btaf525_Supplementary_Data
