# Supplementary material for: Decipher RNA isoform combinations from minigene splicing assays and massive parallel sequencing with MAGIC
Source: Bioinformatics. 2025 Sep 18;41(9):btaf525. doi: 10.1093/bioinformatics/btaf525 (PMC12479391; doi:10.1093/bioinformatics/btaf525)

**Decipher RNA isoform combinations from minigene splicing assays and massive parallel sequencing with MAGIC**

Contents:

[**Figure S1**: Mechanisms of the transcript diversity with the corresponding SOSTAR annotation. Black boxes: exon, black lines: intron, red boxes: exon (or part of exon) skipping, green boxes: novel exon (or part of exon). From (Aucouturier et al., 2024). 2](#_Toc205387673)

[**Figure S2**: Example of the fasta file generated by MAGIC GUI of the construction spanning exons 2 to 9 of RAD51D gene. 3](#_Toc205387674)

[**Figure S3**: Example of a gtf file generated by MAGIC GUI of the construction spanning exons 2 to 9 of RAD51D gene. 4](#_Toc205387675)

[**Figure S4**: Capillary electrophoresis of RT-PCR amplicon of the construction spanning exons 2 to 9 of RAD51D gene. Blue peaks represent the FAM-labeled amplicons and orange peaks the LIZ-1200 size standard. FL: Full length transcript. 5](#_Toc205387676)

**Figure S1**: Mechanisms of the transcript diversity with the corresponding SOSTAR annotation. Black boxes: exon, black lines: intron, red boxes: exon (or part of exon) skipping, green boxes: novel exon (or part of exon). From (Aucouturier et al., 2024).


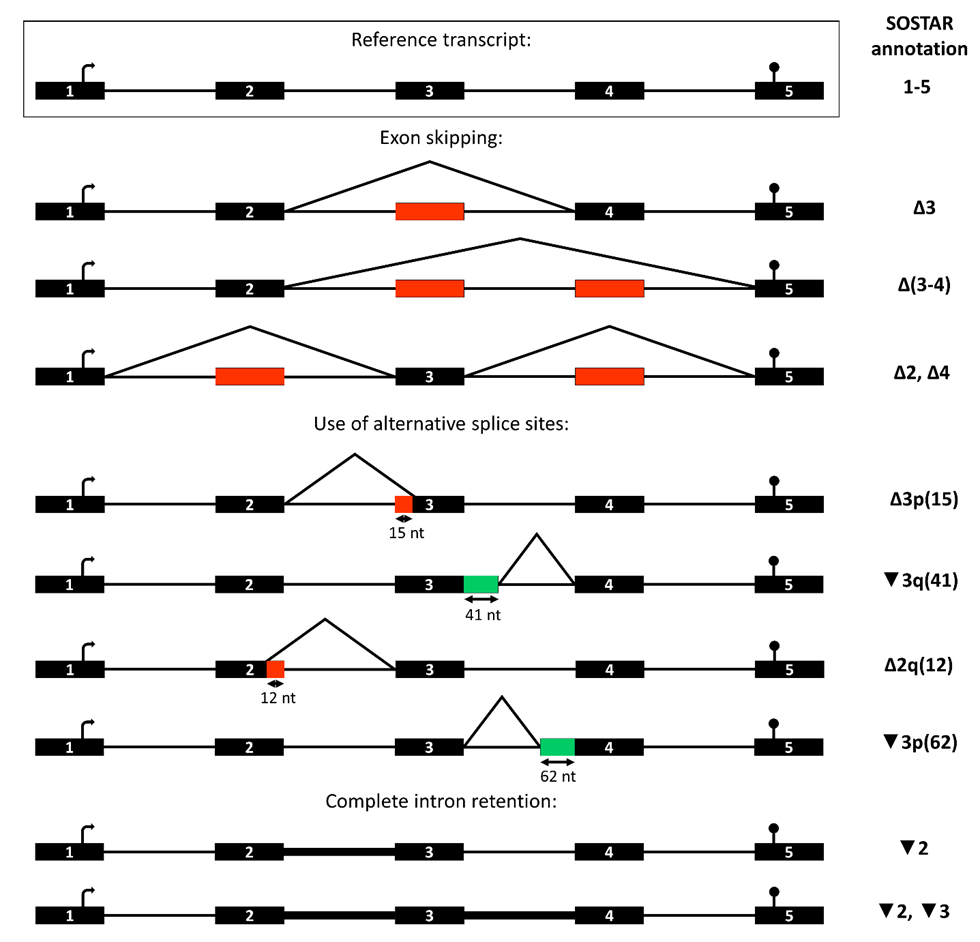


**Figure S2**: Example of the fasta file generated by MAGIC GUI of the construction spanning exons 2 to 9 of RAD51D gene.


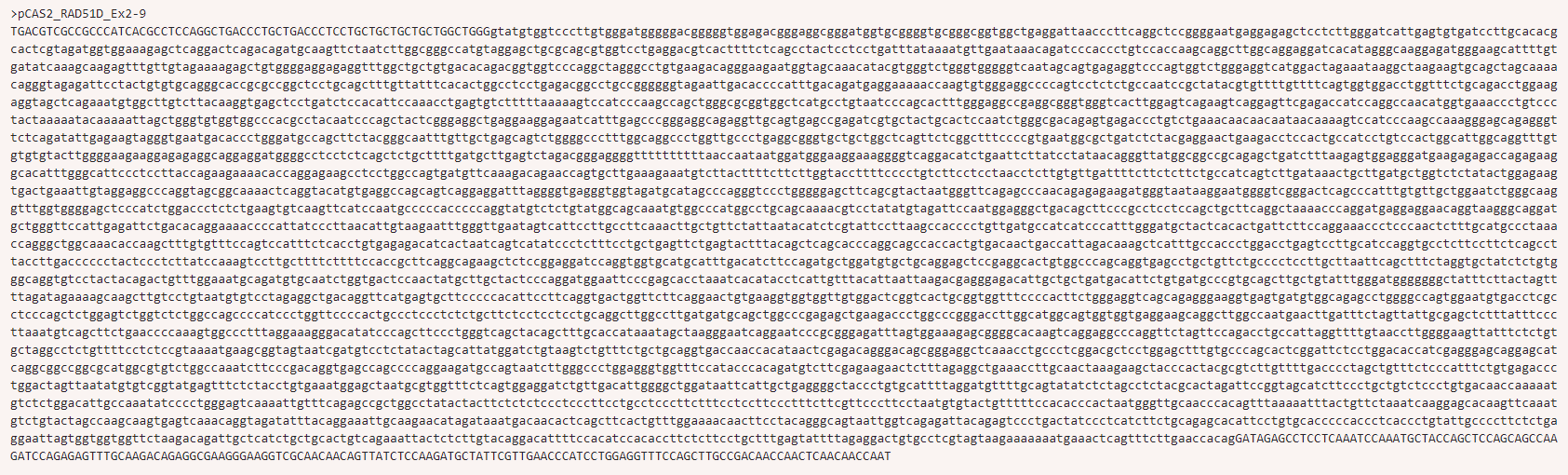


**Figure S3**: Example of a gtf file generated by MAGIC GUI of the construction spanning exons 2 to 9 of RAD51D gene.


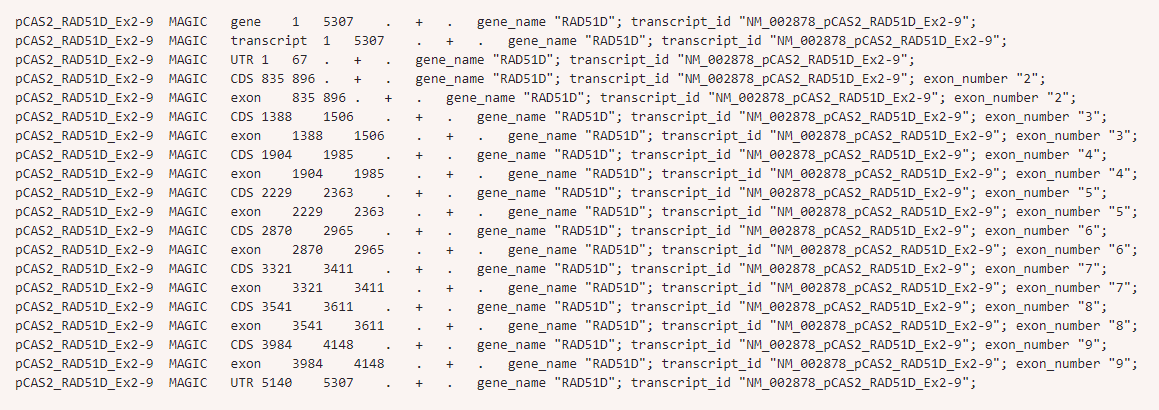


**Figure S4**: Capillary electrophoresis of RT-PCR amplicon of the construction spanning exons 2 to 9 of RAD51D gene. Blue peaks represent the FAM-labeled amplicons and orange peaks the LIZ-1200 size standard. FL: Full length transcript.


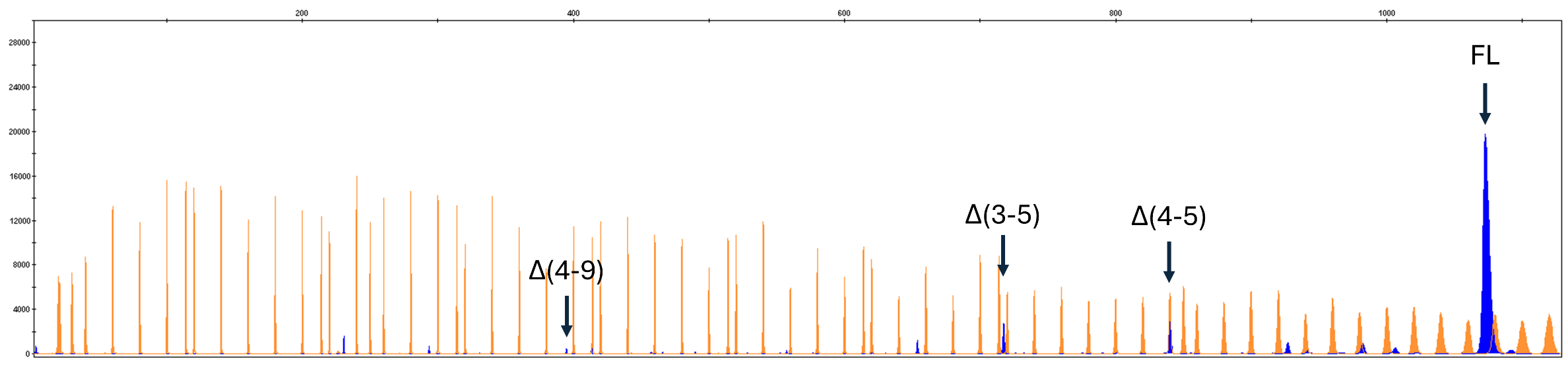

Supplement: btaf525_Supplementary_Data [file btaf525_supplementary_data.docx]
